# Universal Strategy for Reversing Aging and Defects in Graphene Oxide for Highly Conductive Graphene Aerogels

**DOI:** 10.1021/acs.jpcc.3c01534

**Published:** 2023-05-30

**Authors:** Prabhat Kumar, Martin Šilhavík, Zahid Ali Zafar, Jiří Červenka

**Affiliations:** Department of Thin Films and Nanostructures, Institute of Physics of the Czech Academy of Sciences, Cukrovarnická 10/112, Prague 162 00, Czech Republic

## Abstract

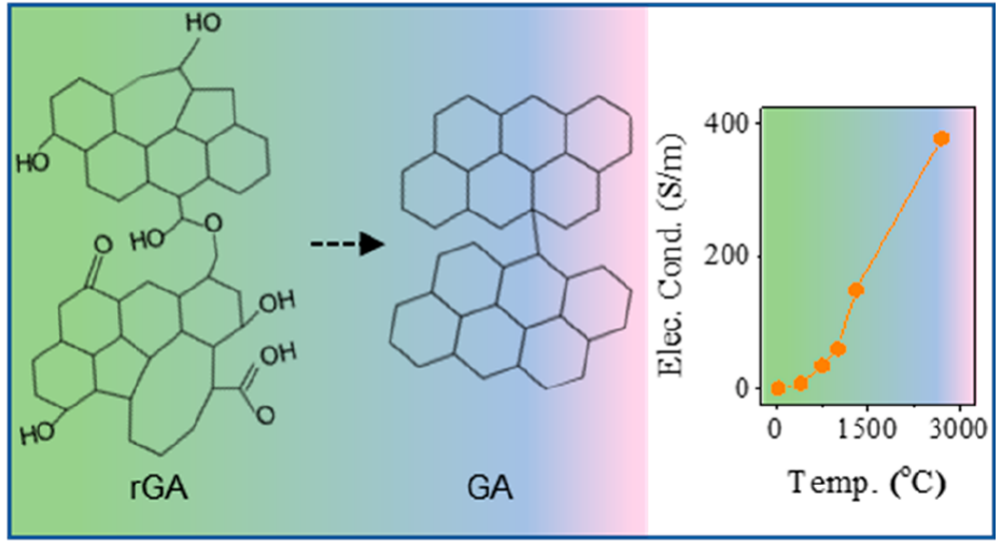

The production of highly stable, defect-free, and electrically conducting 3D graphene structures from graphene oxide precursors is challenging. This is because graphene oxide is a metastable material whose structure and chemistry evolve due to aging. Aging changes the relative composition of oxygen functional groups attached to the graphene oxide and negatively impacts the fabrication and properties of reduced graphene oxide. Here, we report a universal strategy to reverse the aging of graphene oxide precursors using oxygen plasma treatment. This treatment decreases the size of graphene oxide flakes and restores negative zeta potential and suspension stability in water, enabling the fabrication of compact and mechanically stable graphene aerogels using hydrothermal synthesis. Moreover, we employ high-temperature annealing to remove oxygen-containing functionalities and repair the lattice defects in reduced graphene oxide. This method allows obtaining highly electrically conducting graphene aerogels with electrical conductivity of 390 S/m and low defect density. The role of carboxyl, hydroxyl, epoxide, and ketonic oxygen species is thoroughly investigated using X-ray photoelectron and Raman spectroscopies. Our study provides unique insight into the chemical transformations occurring during the aging and thermal reduction of graphene oxide from room temperature up to 2700 °C.

## Introduction

Graphene oxide (GO) is a two-dimensional material constituting individual graphene sheets decorated with various oxygen functional groups on the basal planes and edges.^1^ GO is typically derived from graphite through an oxidation treatment which makes it soluble in water.^2,3^ The solubility of GO in water has enabled the fabrication of more complex three-dimensional (3D) graphene structures and building blocks, such as graphene aerogels, graphene foams, graphene sponges, holey graphene, and graphene frameworks.^3,4^ 3D graphene materials are highly porous and exhibit an extremely high surface area, efficient mass transport, and high electron conduction. They retain some of the exceptional properties of 2D graphene but also exhibit fundamentally new properties.^5−7^ 3D graphene materials have been extensively explored in various macroscopic applications, such as energy storage,^8,9^ electrocatalysts,^10^ sensing devices,^11^ oil and organic solvent absorbers,^12−15^ and flame-retardant materials.^16,17^ However, finding an ideal reduction method for the production of high-quality 3D graphene materials that can fully remove oxygen-containing functionalities and lattice defects from the GO precursors is difficult.^5−7,17^

Several synthesis methods have been developed using GO as a starting material for the preparation of 3D graphene materials (summarized in Table S1).^18−29^ Most of these synthesis methods involved two steps: (i) self-assembly of GO flakes into the 3D structure and (ii) reduction of GO into graphene without restacking. These steps can be performed simultaneously or one after the other. Although the GO-based methods are simple and versatile, there are challenges associated with them.

One of the major issues that all the GO-based synthesis methods suffer from is the low mechanical stability, strength, and electrical conductivity of the resulting 3D graphene materials.^17^ This is because chemically reduced GO materials contain many oxygen functional groups, defects, and nanoscale inhomogeneities. More importantly, graphene layers can restack through weak van der Waals interactions during the synthesis, which together with the increased density of defects leads to a partial or complete loss of the remarkable 2D electronic properties of graphene in the 3D structures.^30,31^

The synthesis of 3D graphene materials also strongly depends on the chemical composition of the starting GO material.^17^ Because GO is not a chemically well-defined material, it can contain variable ratios of carbon, oxygen, and hydrogen, depending on the production method and the type of reaction.^3,4,32,33^ Recently, aging of GO has been observed after long-term storage or exposure to light.^34−36^ The aging results in irreversible changes in the chemical composition of GO after the interaction with the environment.^36,37^ GO was found to degrade even faster upon exposure to light. As GO materials are commonly collected and stored as dry powders or in liquids until further usage, aging poses a severe issue for practical applications and the fabrication of 3D graphene materials. The GO stored in liquid form by making a GO dispersion in water also undergoes changes with time.^38,39^ Therefore, effective mitigation strategies are needed to prevent aging and defects in GO-based 3D graphene materials. Recently it has been shown that plasma treatment can effectively modify the chemical structure and properties of GO materials.^40−42^ However, the effects of plasma treatment have not been investigated on aged GO and 3D graphene synthesis yet.

In this work, we investigate how aging and defects influence the synthesis and resulting properties of 3D graphene aerogels from GO precursors dispersed in water. We analyze the structural and chemical changes of GO caused by aging and during the individual steps of the three-step hydrothermal synthesis of 3D graphene aerogels. We show that the effects of aging on graphene oxide flakes can be reversed by utilizing oxygen plasma. Our strategy is based on controlling the flake sizes and oxygen functional groups attached to the graphene oxide flakes, which helps to self-assemble flakes into a stable and intact 3D graphene hydrogel. Moreover, we investigate the effect of different temperature annealing at 400–2700 °C of the chemically reduced graphene aerogels. Our study provides unique and complex insight (XPS, FTIR, Raman spectroscopy, and zeta potential measurements) into graphene oxide aging and chemical transformations to graphene occurring during the annealing from room temperature up to 2700 °C. We demonstrate that the high-temperature annealing at 2700 °C completely removes all the residual oxygen species and repairs intrinsic structural defects in the materials without affecting their structure. As a result, we obtain highly mechanically stable and conductive graphene aerogels with extremely low defect density. We demonstrate a practical and universal strategy to heal defects and improve the electrical properties of complex 3D graphene structures without affecting their morphology.

## Experimental Section

### Synthesis of Graphene Aerogels

The graphene aerogels were prepared using different kinds of graphene oxide precursors: new GO, aged GO (aGO), and plasma-treated aged GO (pGO). The new GO powder was purchased commercially from XFNano. The GO aging was performed by keeping the GO powder for 12 months in a sealed container to make aGO. Then aGO was exposed to an O_2_ plasma environment for 120 s, at 50 W, process pressure of 50 Pa with 50 sccm of O_2_ gas, and frequency *f* = 8.0 MHz (TESLA Rožnov) to produce pGO.

A GO and deionized (DI) water solution was prepared by mixing 2 mg/mL of the GO powders in DI water. A homogeneous dispersion solution was obtained after ultrasonication for 30 min. Then the solution (30 mL) was sealed in a Teflon-lined autoclave of 50 mL size. The autoclave was heated at 180 °C for 6 h to yield a self-assembled interconnected 3D microporous network of reduced graphene oxide (rGO) hydrogels. The 3D rGO hydrogels were subsequently freeze-dried in a vacuum (2 × 10^–1^ mbar) at −70 °C for 16 h to remove the residual water content. The rGO aerogels before the annealing are termed nonannealed rGA.

The obtained freeze-dried rGA samples were annealed at different temperatures, i.e., 400, 750, 1000, 1300, and 2700 °C, to obtain graphene aerogels (GA). For annealing, a homemade vacuum furnace (see Figure S1) was utilized with a graphite paper heating element that surrounded the annealed samples. All the annealing was performed in the vacuum furnace at 2.3 × 10^–4^ mbar for 30 min. The annealing temperature was monitored by a pyrometer (Optris, model: CSlaser 2MH CF2).

### Material Characterization

Scanning electron microscopy (TESCAN MAIA3) was used to characterize the rGA and GA morphology. The materials were measured by X-ray photoelectron spectroscopy (XPS Kratos Analytical Ltd.) and Raman spectroscopy (Renishaw inVia setup using a 442 nm laser). Zeta potential and flake size measurements were performed with a ZetaNano ZS (Malvern Instruments) device equipped with a He/Ne laser operating at 633 nm as a light source and an avalanche photodiode as a detector. The DLS analysis used in this work is based on the model which considers spherical particles. DC conductivity measurements were performed by linear four-point probe method by applying a constant current of 0.05 mA using a constant current source (Keithley, Models 236 and 237) and measuring the voltage via a multimeter (ProsKit, Model MT-1820). The measurement was done by slightly pressing the sample (≤5% strain) on the top of predeposited electrodes to achieve good electrical contact.

## Results and Discussion

**Figure 1:**
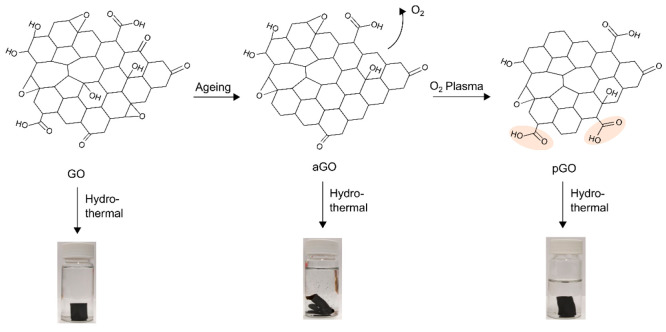
Schematics of the fabrication process and actual photographs of the graphene hydrogels obtained using hydrothermal synthesis from GO, aGO, and pGO as starting materials.

Figure 1 shows the negative effect of the GO aging on the hydrothermal synthesis of 3D graphene aerogels (GA). When producing materials, it is imperative to reproduce them with the most similar properties. Therefore, one of the crucial parts in the hydrothermal synthesis of GA is always to have the same starting GO material, which yields a compact and stable graphene hydrogel after the synthesis.^17^ GO used in this study was purchased from commercial sources. That is why its properties can vary from batch to batch due to aging or different storage conditions. In most cases, the purchased GO enabled formation of a stable hydrogel using hydrothermal synthesis. However, older GO affected by 1 year aging (aGO) did not yield a consolidated graphene hydrogel. Instead, distorted and broken pieces were produced from aGO precursors using the same hydrothermal synthesis parameters (Figure 1). Some newly purchased batches of GO from the supplier have also failed to produce compact cylindrical hydrogels.

We have found that this issue could be overcome by exposing them to oxygen plasma. The plasma-treated aGO (pGO) materials have formed a compact and stable rGO hydrogel using hydrothermal synthesis again. Figure 1 shows that the resultant GA cylinders made of the pGO precursors are similar to those from the fresh GO precursors. This result indicates that the plasma can be effective in modifying the structural and chemical composition of the aged GO to yield stable and mechanically robust graphene hydrogels in hydrothermal synthesis.

### Effects of Aging

**Figure 2:**
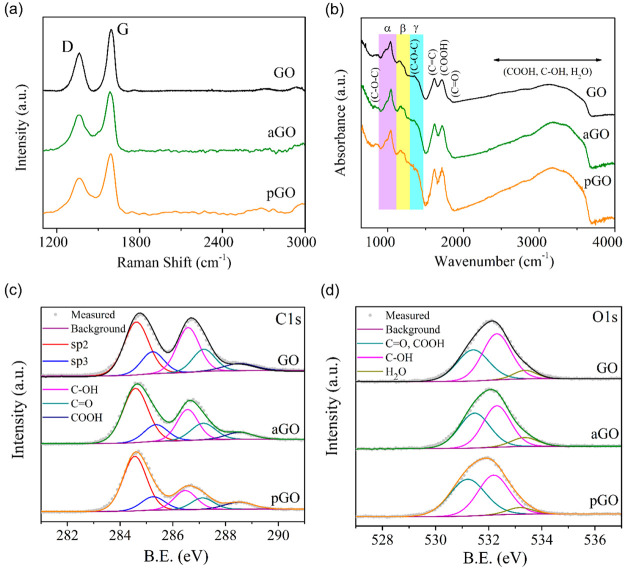
(a) Raman, (b) FTIR, and XPS (c) C 1s and (d) O 1s spectra of GO, aGO, and pGO (GO: new graphene oxide; aGO: aged graphene oxide; pGO: plasma-treated aged graphene oxide).

To understand the effects of the GO aging, a thorough examination of the structure and chemical composition of GO before and after the aging was performed in Figure 2. The new GO and aged aGO samples were analyzed by FTIR, Raman, and XPS. The Raman spectra of the GO and aGO samples (Figure 2a) depict two major peaks at 1368 and 1598 cm^–1^, which are associated with the D and G bands of graphene oxide. The *I*_D_/*I*_G_ ratio of GO and aGO is 0.87 and 0.81, respectively. No major noticeable change can be observed in the Raman spectra of GO compared to aGO. The FTIR absorbance spectra of GO and aGO (Figure 2b) reveal several vibrational peaks. These peaks are ascribed to the vibration modes of carboxyl (COOH at 1615–1725, 995, and 3150 cm^–1^), hydroxyl (C–OH at 2965–3800 and 1135 cm^–1^, including vibrations from COOH and H_2_O), epoxide (C–O–C at 1200–1385 cm^–1^), sp^2^-hybridized (C=C at 1550–1650 cm^–1^), and ketonic species (C=O at 1725–1810 cm^–1^).^34,43^ The main difference between the FTIR spectra of the GO and aGO samples is observed in the relative intensities of the peaks. This observation shows a quantitative difference between the total content of oxygen and oxygen functional groups in the new and aged GO samples. However, due to the overlap of the peaks, quantitative analysis of the chemical changes is difficult and inaccurate from FTIR.^43^ Therefore, quantitative chemical analysis was done using XPS.

XPS analysis of GO and aGO (Figure 2c,d, Table 1, and Tables S2–S4) confirms that the aged samples are slightly chemically different from the fresh GO. The C 1s spectrum of the fresh GO (Figure 2c) after deconvolution can be assigned to five major peaks. The peaks at 284.6 and 285.2 eV correspond to the carbon–carbon bonds with sp^2^ and sp^3^ hybridization, respectively. The remaining three peaks are ascribed to oxygen functional groups. As the peaks of epoxide and hydroxyl groups have similar binding energies,^44^ they are shown as a combination of a single C–OH peak at 286.5 eV. The ketonic, carbonyl, and quinone species (C=O) are located at 287.1 eV, and the carboxyl species (COOH) are at 288.5 eV.^35,45,46^ The corresponding O 1s spectra with the deconvoluted C–O, C=O, and COOH peaks of the GO are shown in Figure 2d.

**Table 1:** Quantitative Analysis of Elements and Functional Groups in Graphene Oxide Using XPS

	concentration (%)							
sample	total C	total O	C–C sp^2^ a	C–C sp^3^ a	C–OH^a^	C=O^a^	COOH^a^	H_2_O^a^
GO	71	29	36	14	28	14	6	2
aGO	72	28	44	13	21	13	6	3
pGO	76	24	51	13	17	10	8	2

a The concentrations were determined from the areas of the C 1s peaks in the fitting analysis.

After the aging, the C 1s and O 1s spectra (Figure 2c,d) depict a slight decrease in the oxygen-functionalized carbon peak in aGO compared to the fresh GO. The elemental analysis shows that the oxygen content is decreased by ∼1% in aGO compared to GO (Table 1). The deconvoluted C 1s and O 1s peaks (Tables S3 and S4) reveal that the aging causes a loss of the oxygen-functionalized carbon, while the relative percentage of the sp^2^ + sp^3^ carbon content increases. A 7% decrease is observed related to the C–OH groups and an ∼1% decrease in the content of C=O in aGO. Overall, the aging results in the loss of oxygen and modification of the oxygen-related functional groups in GO.

### Plasma Treatment

The structural and chemical composition analysis of the plasma-treated aged graphene oxide (pGO) in Figure 2 and Table 1 reveals that oxygen plasma exposure can induce complex changes in aGO. The exposure time has been carefully optimized to ensure no structural damage to the aGO flakes. It can partly reverse some of the changes caused by the aging, but it does not recover the relative content of the oxygen and carbon species back to the original nonaged GO. The Raman spectrum of the pGO sample (Figure 2a) depicts a slight decrease in the *I*_D_/*I*_G_ ratio down to 0.65 compared to the aGO and GO samples and improvement of the crystallinity of the sample. This observation can be explained by the partial removal of defects related to oxygen functional groups with the plasma.^40^ There is no major difference in the FTIR spectrum of pGO with respect to aGO. On the other hand, XPS exhibits notable chemical changes in pGO compared to aGO (Figure 2c,d and Tables S2–S4). The deconvoluted XPS peaks in the C 1s and O 1s spectra show that the absolute oxygen content was decreased by 4% after the plasma treatment. This is mainly reflected in the decrease of hydroxyl (C–OH), epoxide (C–O), and ketonic (C=O) functional groups. However, the relative content of carboxyl (COOH) was increased from 6.1 to 8.3% in pGO compared to aGO (see Table S3). The amount of sp^2^-bonded carbon is also increased by the plasma treatment. Interestingly, the amount of defect-related sp^3^ carbon remains unchanged in the pGO after plasma treatment, which is in line with the Raman observation.

From XPS analysis, the following conclusion can be derived. There are minor compositional changes in graphene oxide caused by the O_2_ plasma treatment. Most of the oxygen species are diminished in the GO samples, except for COOH, which is increased. As the carboxyl groups are negatively charged, this observation suggests that a small increase in the COOH species by plasma is sufficient to recover the highly negative charge of aGO and improve the solubility in water, as demonstrated below. However, it needs to be highlighted that it is challenging to draw any concrete conclusion from the structural, vibrational, and compositional analysis due to the insufficient sensitivity of the spectroscopic techniques to the carbon–oxygen bonds.^3^ This is because, in reduced GO, all carbon atoms in the defective regions are bonded to three neighbors that maintain a planar sp^2^ configuration, making them undetectable by spectroscopic techniques.^32^ Therefore, the GO properties, such as the surface charge and flake size distribution, influenced by the aging and plasma treatment are analyzed in water solutions in the next section.

### Stability of Graphene Oxide Suspensions

**Figure 3:**
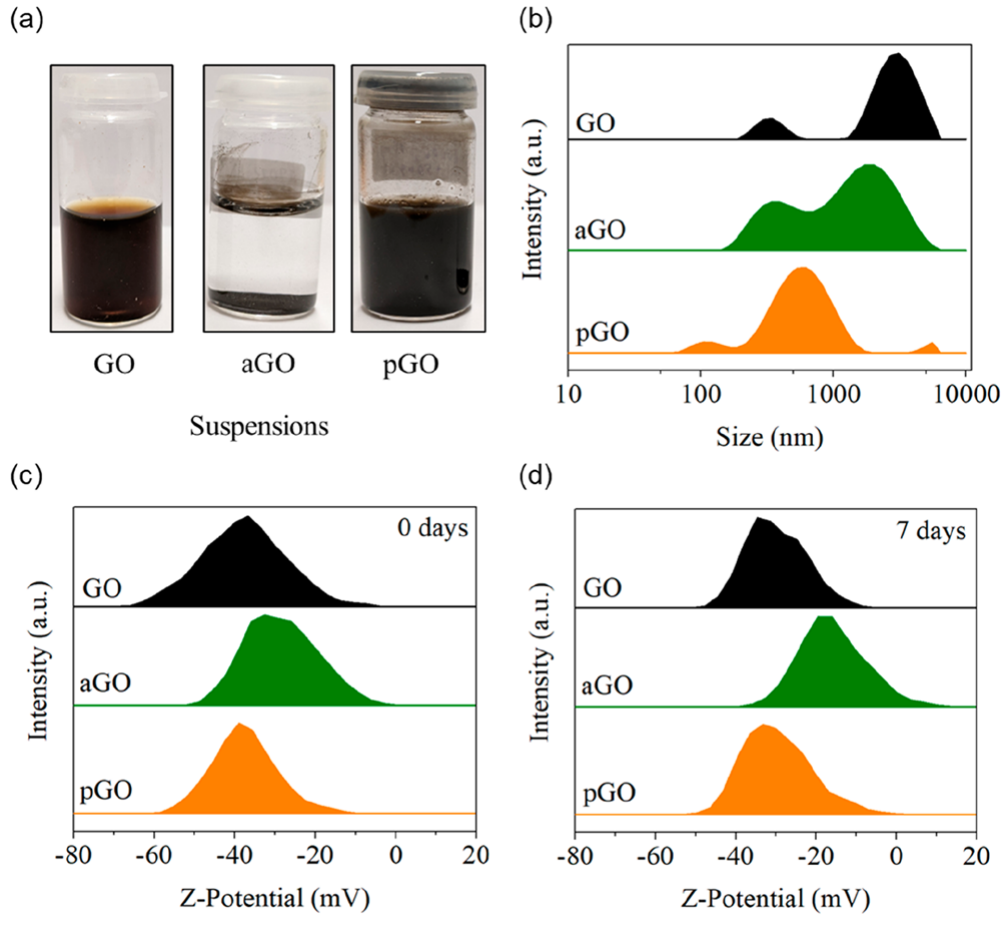
Properties of GO, aGO, and pGO suspensions in water. (a) Photographs of the GO, aGO, and pGO solutions with water after 7 days. (b) Size distribution of the aGO solution immediately after mixing. Zeta potential of (c) the fresh and (d) 7-day-old suspensions.

As the hydrothermal synthesis of GA is started with an aqueous solution of GO, it is necessary to have good colloidal stability of GO suspensions in water for the successful preparation of 3D graphene hydrogels. Figure 3 shows that there is significantly different stability of GO, aGO, and pGO suspensions in water immediately after mixing and after 7 days. The fresh, highly oxidized GO is well-soluble in water (Figure 3a), demonstrating no precipitation at the bottom of the bottle even after 7 days. However, the colloidal stability of the aGO suspensions is lost due to the adverse effects of the GO aging. The plasma-treated pGO flakes show again good dispersibility in water and their suspensions remain stable even after 7 days, like the GO solution.

Dynamic light scattering (DLS) and zeta potential measurements (Figure 3b–d) reveal that the flake size and the zeta potential of the GO, aGO, and pGO suspensions in water are different. The GO sample shows a broad distribution of sizes with two main peaks at ∼3000 and ∼330 nm. The aGO sample shows a slight decrease in the size of the flakes compared with the GO sample. The smaller flake size in aGO is most probably caused by the fact that the majority of the larger flakes get sedimented at the bottom of the flask. It is also possible that the smaller size results from the local chemical modifications and structural rearrangements of the flakes in water. After plasma treatment, a sharp drop in the sizes of flakes is noticed in the pGO suspension (Figure 3b). The pGO suspension demonstrates a broad, intense peak at ∼590 nm and minor peaks at 110 nm and 5.5 μm (Figure 3b). This result clearly shows that the plasma treatment reduced the sizes of the GO flakes in pGO. The reactive and energetic species in the plasma had sufficient energy to break the GO flakes into smaller sizes. A similar phenomenon has been recently observed during the ultrasonication of the GO solution.^47^

The zeta potential of the GO solutions was measured right after mixing GO with water and a week after the preparation (Figure 3c,d). Generally, the zeta potential smaller than −30 mV is considered sufficient for maintaining good colloidal stability of GO solutions in neutral pH aqueous solutions.^48^ The fresh GO solution exhibits a broad distribution of zeta potentials with peak boundaries from −66 to −7 mV and a peak center at −39 mV (Figure 3c). After a week, the zeta potential peak center of the GO solution shifted down to −31 mV (Figure 3d). The aGO solution measured right after mixing it with water also shows a broad zeta potential distribution. The peak boundaries are in the range of from −50 to −2 mV and a peak center at −30 mV. A week-old aGO suspension demonstrates a peak center upshift to −18 mV, which can be associated with the observed degradation of the aGO colloidal stability. The plasma-treated pGO recovers good colloidal stability by downshifting the zeta potential and restoring the negative zeta potential of the solution to similar values as the fresh GO solution. The measured zeta potential of the pGO solution has slightly narrower peak boundaries from −58 to −12 mV and a peak center at −39 mV. After a week, the zeta potential peak center of the pGO solution slightly upshifted to −31 mV but still remained comparable to the fresh GO suspension. The improvement in the surface charge and stability of pGO in water is attributed to the smaller flake size and restoration of the ratios of carboxylic and hydroxyl groups, as indicated in the XPS analysis (Figure 2 and Table 1). The functional groups help obtain more negative zeta potential in water by ionizing oxygen-containing functional groups into negatively charged radicals.^47,49^

The observed chemical changes and deoxidation of the aGO materials can partly explain the instability of the aGO dispersions in water. Recent studies have suggested that a strong electrostatic repulsion between GO flakes is more important for the formation of a stable GO solution than the simple hydrophilicity of GO, as previously presumed.^48,50^ In this regard, GO can be perceived as an amphiphile with hydrophilic and negatively charged edges and a sizable part of a more hydrophobic and less charged basal plane. GO sheets have phenol, hydroxyl, and epoxide groups on the basal plane and carboxylic acid at the edges.^33,48^ The basal plane of GO also consists of hydrophobic polyaromatic islands of unoxidized benzene rings.^51,52^ Our results show that the negatively charged carboxylic groups at the edges of GO flakes play a key role in forming a stable dispersion in water, which is in line with previous studies.^47,49^ The plasma treatment reverses aging by altering the relative content of oxygen groups, resulting in a smaller size of graphene oxide flakes in water. The plasma decreased the flake size in pGO, resulting in higher edge-to-area ratios. As the density of the functional groups is higher at the flake edges,^53,54^ a higher electrostatic repulsion between flakes and thus better colloidal stability is attained for pGO than aGO in water. This treatment restores the negative zeta potential and the stability of the water suspension, allowing hydrothermal synthesis to produce mechanically stable and intact hydrogels (as shown in Figure 1). On the other hand, the aging causes the desorption of oxygen species and relative increment in the hydrophobic unoxidized graphene areas on the base plane of aGO. Therefore, the aging destabilizes the colloidal stability of aGO suspensions and negatively impacts the hydrothermal synthesis of 3D reduced graphene oxide structures.

### Defects Removal from Reduced Graphene Oxide Aerogel

**Figure 4:**
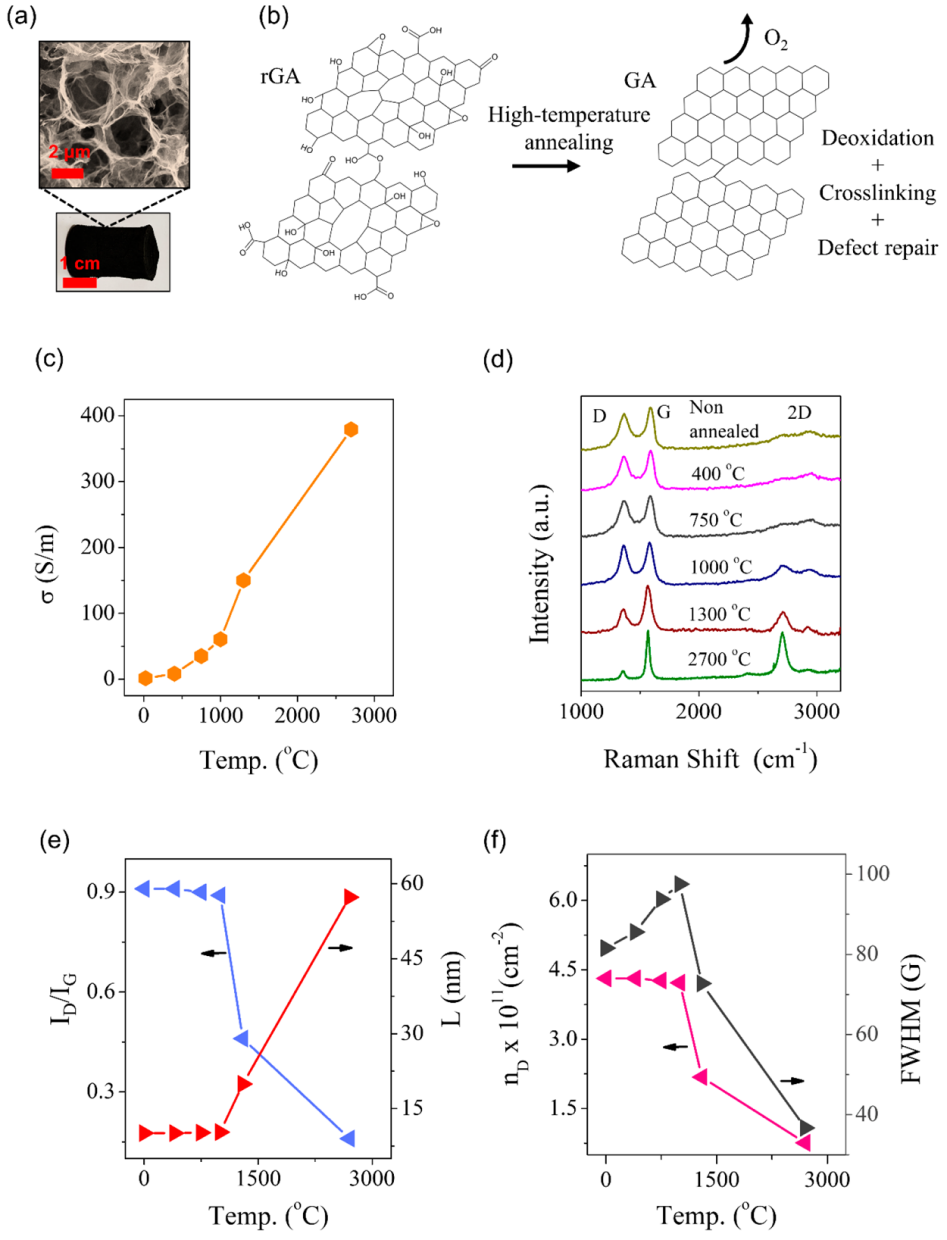
(a) SEM and optical images of a graphene aerogel and (b) schematics of the chemical changes caused by high-temperature annealing, which leads to a removal of oxygen functional groups and structural defects and covalent cross-linking. (c) Electrical conductivity of graphene aerogels as a function of annealing temperature using a four-probe method. (d) Raman spectra of GA annealed at different temperatures. (e) *I*_D_/*I*_G_ ratio and crystallite size analysis as a function of annealing temperature. (f) Density of defects and FWHM of the G peak as a function of annealing temperature.

Another challenge in the synthesis of complex 3D structures of graphene from GO precursors is defects. Defects usually worsen the physical properties of graphene.^17,31,55^ Most crystallographic defects in reduced graphene oxide aerogels (rGA) are inherited from the starting GO material because it contains a high density of sp^3^-hybridized carbon bonds due to the adsorbed oxygen species.^31^ Some intrinsic defects, including lattice/topological and edge defects, are then produced during the ultrasonication and reduction of GO during the hydrothermal synthesis of rGA due to the loss of oxygen functional groups.^56^ 3D porous graphene structures made of interconnected graphene sheets also contain other types of longer-range defects, such as pore defects, cracks, and lack-of-fusion pore structures (Figure 4a,b). These longer-range defects are created during hydrothermal synthesis owing to inhomogeneities and the electrostatic repulsion between flakes in the water dispersions. All of these defects harm the mechanical properties of the aerogels.^17,57,58^ Therefore, the freshly prepared rGA is fragile and has low structural stability.^59^ The rGA also has relatively poor electrical conductivity.^17,56^

In Figure 4, we show the effect of high-temperature annealing of rGA on the electrical conductivity and Raman spectra. The rGA samples were gradually annealed at 400, 750, 1000, 1300, and 2700 °C in a vacuum. The annealed rGA samples are termed graphene aerogels (GA). The SEM analysis of the annealed GA shows no change in the porous structure of the materials after annealing (Figure S2). The high-temperature annealing can significantly improve the electrical conductivity (σ) of the aerogels (Figure 4c). It also leads to the covalent cross-linking of the individual flakes in the pore walls (Figure 4b), which has a prominent effect on the mechanical properties and strength of the aerogels. More detailed information about the mechanical properties of the obtained graphene aerogels can be found in our previous works.^11,16,58^ At lower temperatures (≤1000 °C), the change in σ is low due to the presence of numerous oxygen-related functional groups. The electrical conductivity increases significantly at high temperatures, demonstrating σ of ∼390 S/m at 2700 °C. This electrical conductivity is one of the highest that was reported in the literature for graphene aerogels.^17^

The increase of the electrical conductivity with increasing annealing temperature is well correlated to the observed improvement of the graphene crystallinity measured by Raman spectroscopy (Figure 4d–f). The Raman spectra of the annealed GA samples demonstrate an increase of the 2D band, a decrease of the D band, and a sharpening of the G band with increasing temperature. The D peak is most relevant for determining the structural disorder in graphene, and its intensity tends to grow with a higher number of structural defects.^60^ The ratio of the intensity of the D to G peaks can be used for the determination of the defect density^61^ (*n*_D_) and crystallite size (*L*_a_) using the equation defined by Cancado et al.^62^ For the nonannealed rGA, the crystallite size is found to be around 10 nm, and the density of defects is roughly 4.3 × 10^11^ cm^–2^ (Figure 4e,f). After annealing, there is observed a significant decrease in the defect density and an increase in the crystallite size (Figure 4e,f). The D to G peak ratio does not change when the rGO is annealed at temperatures lower than 1000 °C. Therefore, the crystallite size and defect density remain almost constant. A more pronounced decrease in the defect density is observed along with the broadening of the G peak after exceeding 1300 °C. The best crystal quality graphene and the lowest density of defects of all the samples are achieved in the GA sample annealed to 2700 °C. This extreme temperature annealing results in a 6 times decrease in the defect density and, at the same time, almost a 6 times increase in the crystal size compared with the nonannealed rGO.

**Figure 5:**
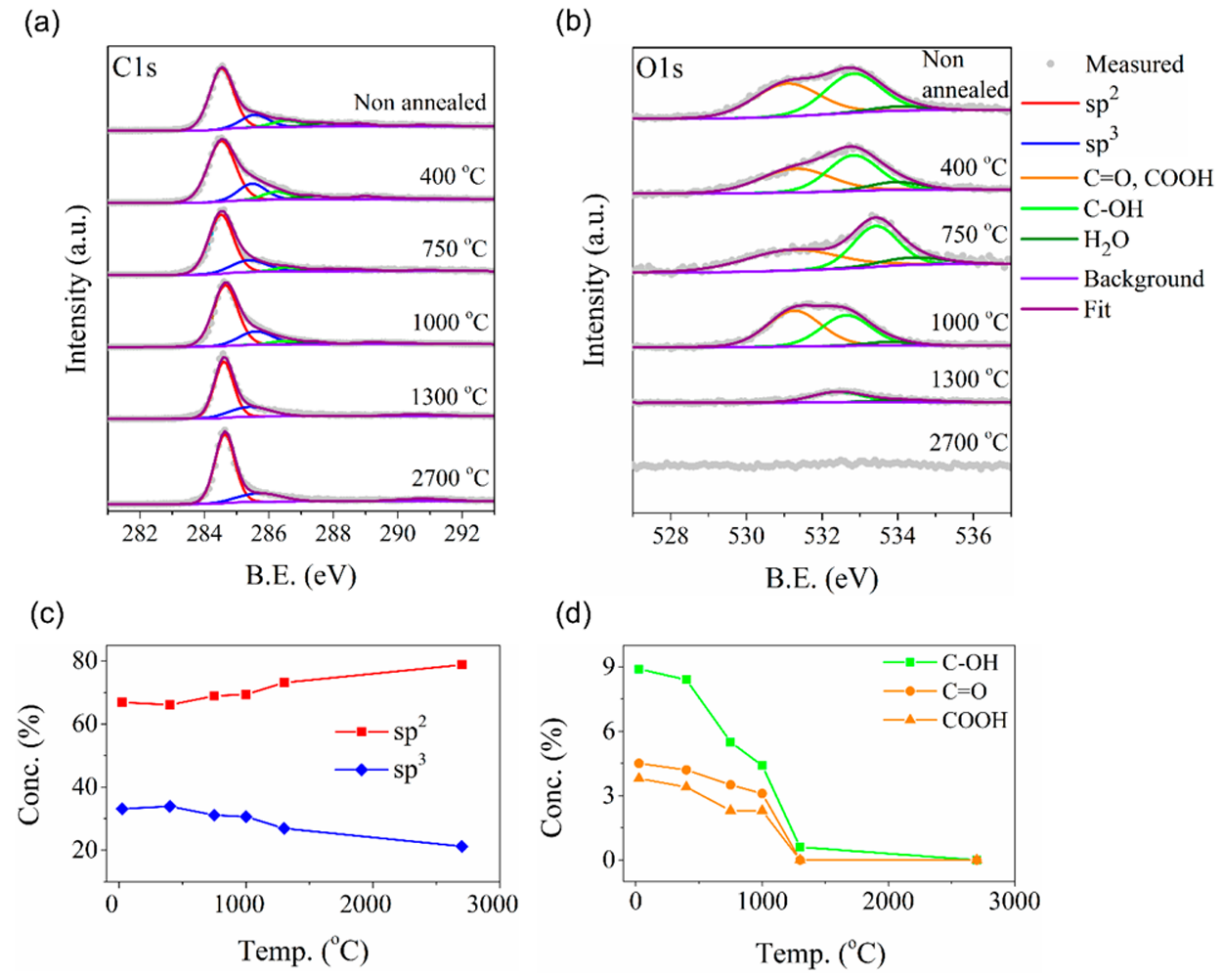
XPS analysis of a reduced graphene oxide aerogel as a function of annealing temperature. (a) C 1s and (b) O 1s spectra of GA annealed at different temperatures. Concentrations of (c) sp^2^ and sp^3^ carbon and (d) different oxygen species in the annealed GA samples. The sp^3^ carbon includes both structural and functional groups.

XPS analysis of the annealed GA samples at temperatures of 400–2700 °C (Figure 5) reveals significant chemical changes compared with the rGA sample before annealing. The XPS C 1s and O 1s spectra of the nonannealed rGA and GA samples are shown in Figure 5a,b, and the corresponding deconvoluted spectra are shown in Figures S3 and S4. The rGO aerogel before annealing depicts C–C (sp^2^) and C=C (sp^3^) hybridized carbon atoms along with several carbon–oxygen functional groups, such as C–O (epoxides and hydroxyl, 286.6 eV), C=O (carbonyl, 287.6 eV), and O–C=O (carboxyl, 288.9 eV).^63−66^ The rGA sample is composed of 89% of carbon and 11% of oxygen. Annealing of the GA at 400 °C shows almost no change in the composition. Once the annealing temperature is increased to 750 °C, the relative content of carbon is increased to 96% and oxygen is decreased to 4%. When the aerogel is further annealed at 1000 °C, the carbon and oxygen content remains almost the same as in the GA annealed at 750 °C. A significant reduction of oxygen is observed after 1300 °C annealing. The GA sample annealed at 1300 °C has >99.4% of carbon and <0.6% of oxygen content. The oxygen is completely removed from the sample when the GA is annealed at 2700 °C (Table S5). After this high-temperature annealing, a highly mechanically stable and intact graphene aerogel is obtained (Figure 4a), as reported in our previous works.^11,16,58^

It is well-known that the oxygen species bonded to graphene have different binding energies.^43,67,68^ Therefore, different oxygen species are removed from the graphene at different temperatures during annealing. A previous study by Acik et al. has reported that the theoretical binding energy for oxygen species desorption ranges from 1.5 to 8 eV.^43^ Hydroxyl groups desorb at 1.5 eV, epoxide at 3.1 eV, carboxyl at 5.8 eV, ketonic at 8.0 eV, and aggregated cyclic edge ether (−O−) at 9.1 eV. The experimentally obtained values from our XPS measurements are in line with this sequence of the theoretical binding energies. The amounts of different oxygen species at a specific annealing temperature in the GA samples determined from XPS are summarized in Tables S6 and S7. The experiments show that hydroxyl groups are removed first. As a result, the GA sample annealed to 1000 °C contains mainly carboxyl and ketonic species.^69^ After 1300 °C annealing, all the remaining oxygen species are removed from the sample.^70−72^ As no more oxygen functional groups are bound to the GA samples at temperatures above 1300 °C, the observed increase of the sp^2^ carbon content in GA between 1300 and 2700 °C (Figure 5c,d) can be entirely attributed to the defect removal. This result shows that 2700 °C annealing can repair some of the crystallographic defects in GA and improve its electrical properties.

## Conclusions

In this work, we investigated the effects of the aging and deoxidation of graphene oxide in the synthesis of complex 3D graphene aerogel structures. We also demonstrated a universal strategy to reverse the aging and remove defects using O_2_ plasma and high-temperature annealing. This strategy allowed us to improve the repeatability of the synthesis of graphene aerogels from aged GO precursors and form highly electrical conducting and stable graphene aerogels with an electrical conductivity of ∼390 S/m. It is found that the aging of GO changes the relative composition of oxygen functional groups, making aGO difficult to disperse in water and form stable reduced graphene oxide aerogels. We showed that the O_2_ plasma could restore good solubility in water by changing the relative content of oxygen groups and decreasing flake sizes in aged GO powders. Moreover, we investigated the effect of different temperature annealing on the removal of the residual oxygen species and defects from reduced graphene oxide aerogels. The low temperature (≤400 °C) annealing was able to remove only hydroxyl species from the aerogels. The complete removal of oxygen species was achieved at temperatures above 1300 °C. Furthermore, it is observed that even after the complete removal of oxygen from GA, the electrical conductivity is still limited due to the presence of intrinsic structural defects. The crystallographic defects can be up to a large extent repaired by heating the aerogels at extremely high temperatures (≥2700 °C). The high-temperature annealing is found as an effective strategy to heal defects and improve the electrical properties of complex 3D graphene structures without affecting their morphology.

## Supporting Information Available

Figures S1: actual photograph of a homemade high-temperature vacuum furnace; Figure S2: scanning electron micrographs of nonannealed and annealed GA; Figures S3 and S4: XPS relative composition analysis of a graphene aerogel annealed at different temperatures; Table S1: various methods for the preparation of the 3D graphene aerogel; Tables S2–S4: XPS elemental analysis of graphene oxide, aged graphene oxide, and plasma treated graphene oxide samples; Tables S5–S7: XPS elemental analysis of graphene aerogels annealed at different temperatures (PDF)
