# Supplementary material for: Universal Strategy for Reversing Aging and Defects in Graphene Oxide for Highly Conductive Graphene Aerogels
Source: J Phys Chem C Nanomater Interfaces. 2023 May 30;127(22):10599–608. doi: 10.1021/acs.jpcc.3c01534 (PMC10258840; doi:10.1021/acs.jpcc.3c01534)
Supplement: Supplementary file 1 — jp3c01534_si_001.pdf [file jp3c01534_si_001.pdf]

Supporting Information

Universal Strategy for Reversing Aging and Defects in Graphene Oxide for Highly Conductive Graphene Aerogels

Prabhat Kumar[†], Martin Šilhavík[†], Zahid Ali Zafar[†], and Jiří Červenka[†*]
[†]Department of Thin Films and Nanostructures, Institute of Physics of the Czech Academy of Sciences, Cukrovarnická 10/112, Prague - 162 00, Czech Republic
**Email: cervenka@fzu.cz*

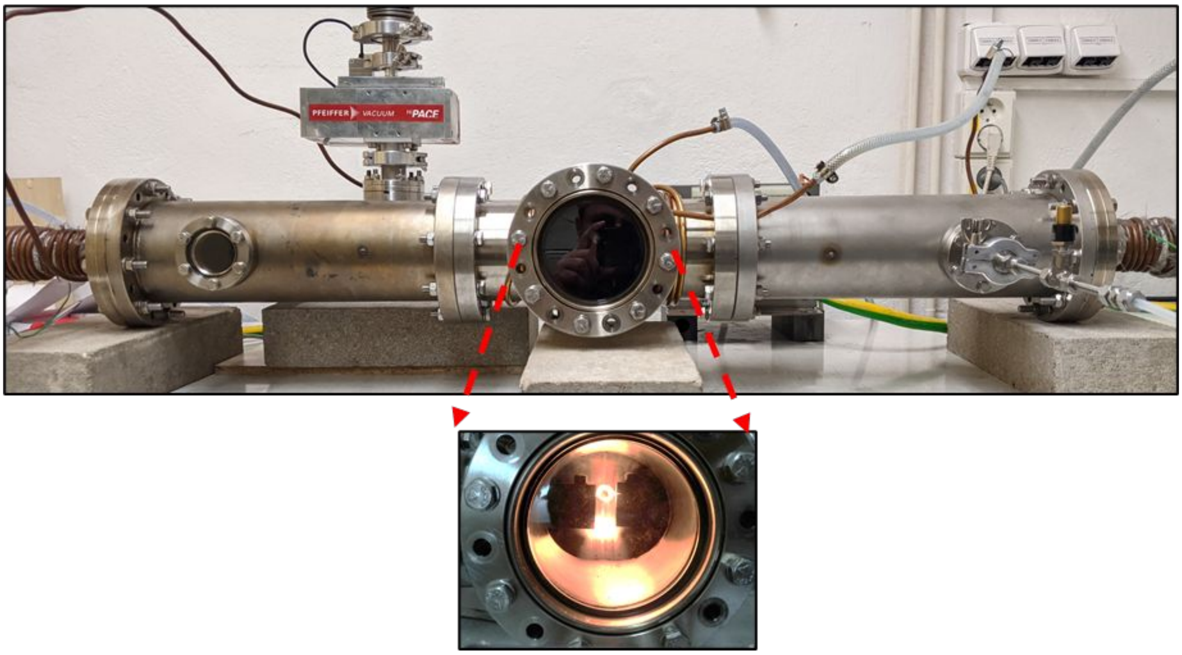

Figure S1: Actual photograph of a home-made high-temperature vacuum furnace.

Table S1: Various methods for the preparation of the 3D graphene aerogel.

3D graphene	Method	Ref
Graphene network	CVD growing on Ni foam template and removal of the template subsequently	1
Graphene foam	Dip-coating of GO solution on Ni foam template, subsequent removal of Ni foam	2
Graphene foam	CVD-growing graphene on Ni foam template and remove the template	3
Graphene foam	CVD-growing graphene on Ni foam template and remove the template	4
Graphene foam	Dip-coating of GO on PU foam template and subsequent pyrolysis of PU template	5
Graphene foam	Dip-coating of GO on PU foam template and subsequent<br>burning of PU template	6
Graphene foam	Hydrothermal reaction of GO/EDA, freeze-drying, and<br>Microwave irradiation	7
Graphene foam	Laser induced Graphene foam	8
Graphene sponge	Modified hydrothermal reaction	9
Graphene cellular network	Emulsion of GO with oil droplet, unidirectional freeze- drying, and thermal reduction	10
Anisotropic cellular graphene	Reduce giant graphene oxide (GGO) with ascorbic acid, followed by directional freeze- drying	11
Cellular graphene	Reduce GGO with ascorbic acid, followed by directional freeze- drying and thermal annealing	12
Pristine cellular graphene	Room-temperature freeze gelation	13
Graphene cellular-based monolith	Partially reduce GO, unidirectional freeze-drying/thawing, and further reduction	14
3D-printed periodic graphene foam	3D printing R-F crosslinked GO solution	15
Foam-like graphene monolith	Emulsion of GO and oil droplet template, hydrothermal<br>reduction, and annealing	16
Macroporous graphene monolith	Hydrothermal synthesis with hexane droplet as soft template	17
Air-bubbled graphene foam	Air-bubbled template, freezing-drying and annealing	18
Anisotropic graphene monolith	Cellulose-templated, dip-coating, and thermal annealing	19

Graphene/Platinum/Nafion Hybrids	Ice templating	[20]
Porous graphene oxide frameworks	Solvothermal reactions	[21]
Three-Dimensional Graphene Monoliths	sp^2-cross-linked	[22]
Holey Graphene	Microscopic engineering	[23]

Table S2: XPS elemental analysis of GO, aGO and pGO samples.

Sample / Element	GO	aGO	pGO
C %	71	72	76
O %	29	28	24
Total %	100	100	100

Table S3: XPS C 1s peak analysis of carbon present in GO, aGO and pGO samples.

Sample	sp^2	sp^3	C-OH	C=O	COOH
**GO**					
Position	284.6	285.3	286.6	287.3	288.5
FWHM	1.1	1.1	1.1	1.1	1.3
%	37.1	14.0	28.5	14.1	6.3
**aGO**					
Position	284.6	285.3	286.5	287.1	288.5
FWHM	1.1	1	1	1.1	1.2
%	45.3	13.1	21.8	13.7	6.1
**pGO**					
Position	284.6	285.3	286.5	287.2	288.5
FWHM	1.1	1.1	1	1	1.3
%	51.5	12.7	17.2	10.3	8.3

Table S4: XPS O 1s peak analysis of oxygen present in GO, aGO and pGO samples.

**Sample**	**C=O + COOH**	**C-OH**	**H_2O**
**GO**			
Position	531.4	532.3	533.4
FWHM	1.4	1.3	1
%	39.9	52.4	7.7
**aGO**			
Position	531.5	532.4	533.5
FWHM	1.2	1.2	1.1
%	43.8	46.4	9.8
**pGO**			
Position	531.2	532.3	533.3
FWHM	1.4	1.5	1.2
%	48.3	45.5	6.2

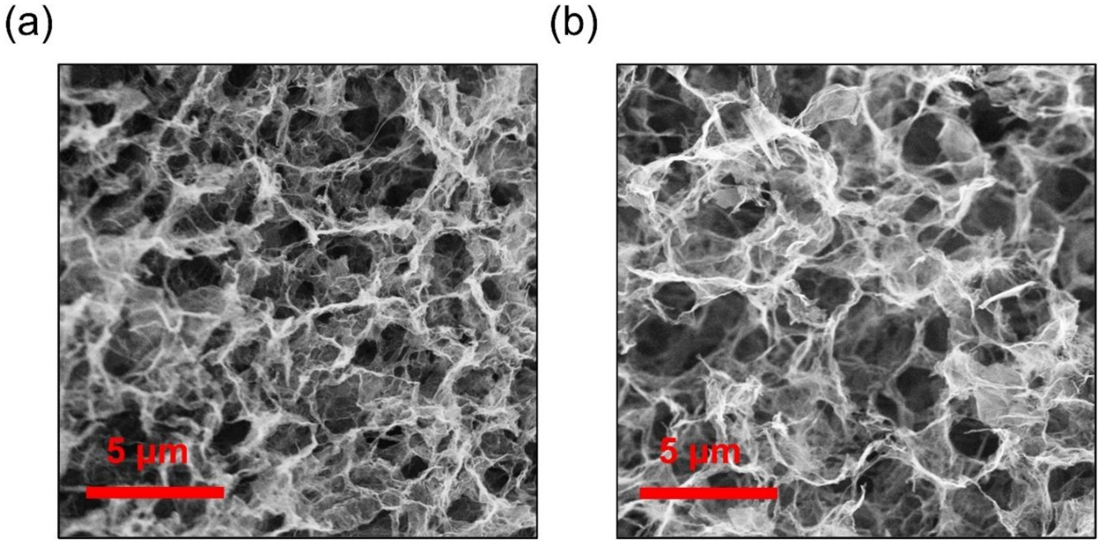

Figure S2: Scanning electron micrographs of (a) non-annealed (b) annealed GA.

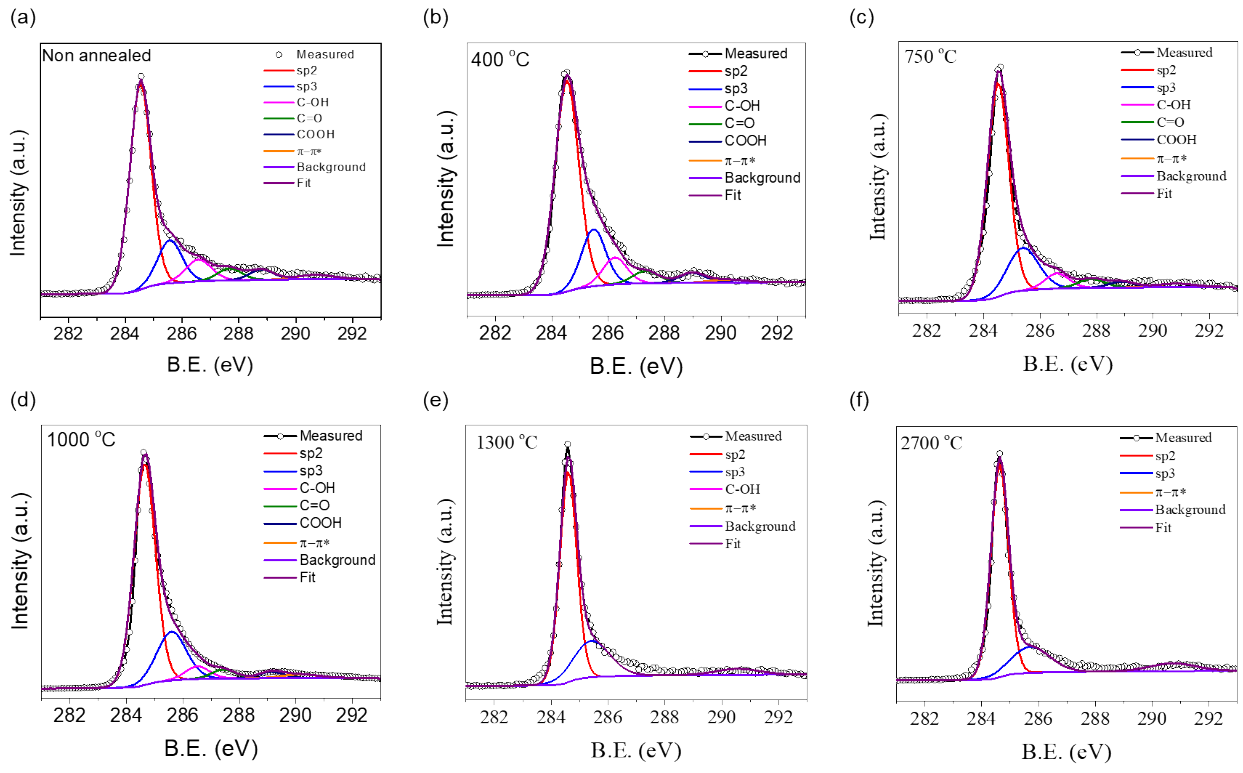

Figure S3: XPS C1s spectra with deconvoluted peaks of GA annealed at different temperatures.

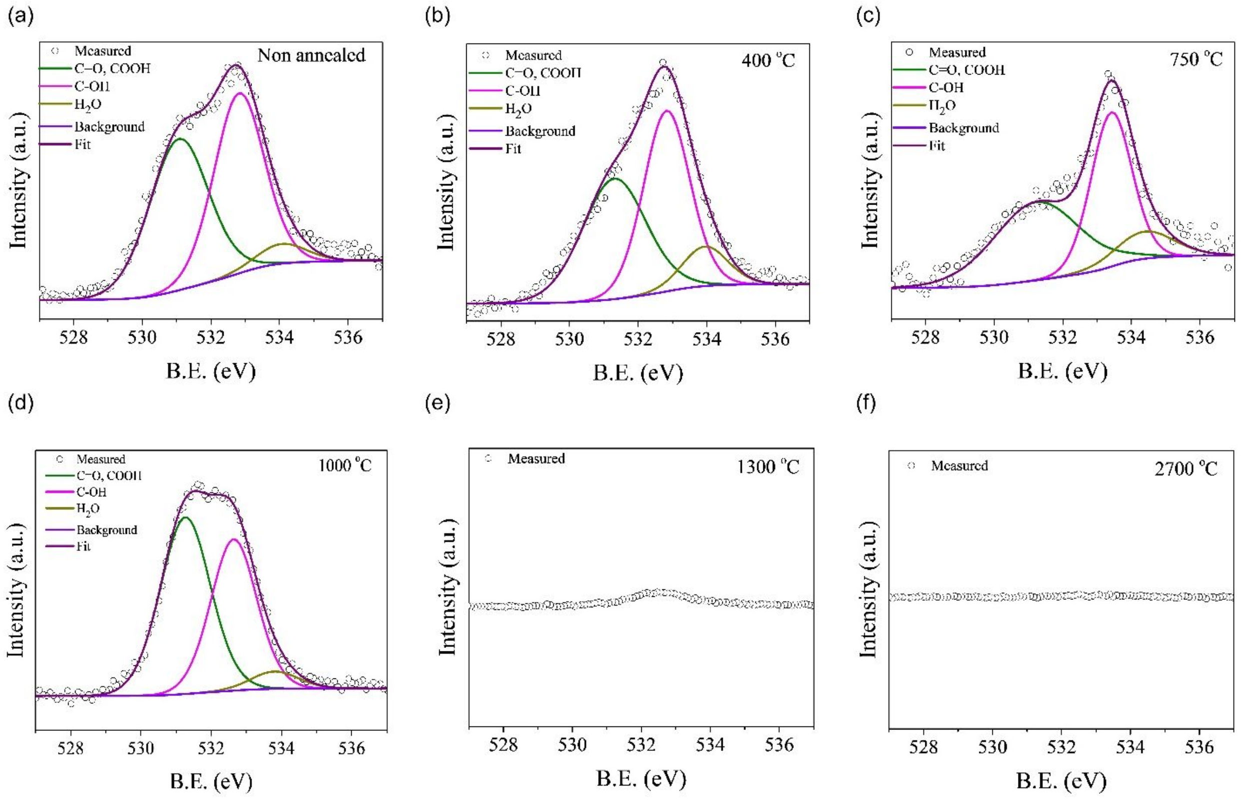

Figure S4: XPS O1s spectra with deconvoluted peaks of GA annealed at different temperatures.

Table S5: XPS elemental analysis of GA as a function of annealing temperatures.

Sample / Element	non annealed	400 °C	750 °C	1000 °C	1300 °C	2700 °C
C %	89	89.8	95.8	96	99.4	100
O %	11	10.2	4.2	4	0.6	0
Total %	100	100	100	100	100	100

Table S6: XPS C1s peak analysis of GA as a function of annealing temperatures.

**Sample**	**sp^2**	**sp^3**	**C-OH**	**C=O**	**COOH**	**π-π***
**non annealed**						
Position	284.6	285.5	286.5	287.6	288.8	290.5
FWHM	0.9	1	1	1	1	1.5
%	66.9	15.9	8.9	4.5	3.8	
**400 °C**						
Position	284.6	285.5	286.3	287.3	288.9	290.3
FWHM	1	1	1	1.1	1.1	1.5
%	66.1	17.9	8.4	4.2	3.4	
**750 °C**						
Position	284.5	285.4	286.6	287.8	288.9	290.8
FWHM	0.9	1.2	1	1.1	1.1	1.5
%	68.9	19.8	5.5	3.5	2.3	
**1000 °C**						
Position	284.6	285.6	286.5	287.5	289.2	290.2
FWHM	0.9	1.2	1	1	1	1.5
%	69.4	20.8	4.4	3.1	2.3	
**1300 °C**						
Position	284.6	285.4				290.4
FWHM	0.7	1.1				1.8
%	73.03	26.97	≤ 1			
**2700 °C**						
Position	284.6	285.7				290.7
FWHM	0.7	1.5				1.8
%	78.8	21.2				

Table S7: XPS O 1s peak analysis of GA as a function of annealing temperatures.

**Sample**	**C=O + COOH**	**C-OH**	**H_2O**
**non annealed**			
Position	531.1	532.8	533.9
FWHM	1.9	1.8	1.8
%	47.1	47.7	5.2
**400 °C**			
Position	531.1	532.7	533.6
FWHM	1.9	1.6	1.7
%	43.1	47.8	9.1
**750 °C**			
Position	531.0	533.2	533.9
FWHM	2.9	1.4	1.9
%	45.8	43.3	10.9
**1000 °C**			
Position	531.2	532.6	533.8
FWHM	1.5	1.6	1.6
%	53.3	41.8	4.9
**1300 °C**	-	532.5	533.9
FWHM		1.6	1.9
%		78.2	21.8
**2700 °C**	-	-	-

References

(1) Chen, Z.; Ren, W.; Gao, L.; Liu, B.; Pei, S.; Cheng, H.-M. Three-Dimensional Flexible and Conductive Interconnected Graphene Networks Grown by Chemical Vapour Deposition. *Nat. Mater.* **2011**, *10* (6), 424–428. https://doi.org/10.1038/nmat3001.

(2) Samad, Y. A.; Li, Y.; Alhassan, S. M.; Liao, K. Novel Graphene Foam Composite with Adjustable Sensitivity for Sensor Applications. *ACS Appl. Mater. Interfaces* **2015**, *7* (17), 9195–9202. https://doi.org/10.1021/acsami.5b01608.

(3) Reddy, S. K.; Ferry, D. B.; Misra, A. Highly Compressible Behavior of Polymer Mediated Three-Dimensional Network of Graphene Foam. *RSC Adv.* **2014**, *4* (91), 50074–50080. https://doi.org/10.1039/C4RA08321K.

(4) Nieto, A.; Boesl, B.; Agarwal, A. Multi-Scale Intrinsic Deformation Mechanisms of 3D Graphene Foam. *Carbon N. Y.* **2015**, *85*, 299–308.

https://doi.org/10.1016/j.carbon.2015.01.003.
(5) Samad, Y. A.; Li, Y.; Schiffer, A.; Alhassan, S. M.; Liao, K. Graphene Foam Developed with a Novel Two-Step Technique for Low and High Strains and Pressure-Sensing Applications. *Small* **2015**, *11* (20), 2380–2385. https://doi.org/10.1002/smll.201403532.
(6) Du, X.; Liu, H.-Y.; Mai, Y.-W. Ultrafast Synthesis of Multifunctional N-Doped Graphene Foam in an Ethanol Flame. *ACS Nano* **2016**, *10* (1), 453–462. https://doi.org/10.1021/acsnano.5b05373.
(7) Hu, H.; Zhao, Z.; Wan, W.; Gogotsi, Y.; Qiu, J. Ultralight and Highly Compressible Graphene Aerogels. *Adv. Mater.* **2013**, *25* (15), 2219–2223. https://doi.org/10.1002/adma.201204530.
(8) Sha, J.; Li, Y.; Villegas Salvatierra, R.; Wang, T.; Dong, P.; Ji, Y.; Lee, S.-K.; Zhang, C.; Zhang, J.; Smith, R. H.; Ajayan, P. M.; Lou, J.; Zhao, N.; Tour, J. M. Three-Dimensional Printed Graphene Foams. *ACS Nano* **2017**, *11* (7), 6860–6867. https://doi.org/10.1021/acsnano.7b01987.
(9) Wu, Y.; Yi, N.; Huang, L.; Zhang, T.; Fang, S.; Chang, H.; Li, N.; Oh, J.; Lee, J. A.; Kozlov, M.; Chipara, A. C.; Terrones, H.; Xiao, P.; Long, G.; Huang, Y.; Zhang, F.; Zhang, L.; Lepró, X.; Haines, C.; Lima, M. D.; Lopez, N. P.; Rajukumar, L. P.; Elias, A. L.; Feng, S.; Kim, S. J.; Narayanan, N. T.; Ajayan, P. M.; Terrones, M.; Aliev, A.; Chu, P.; Zhang, Z.; Baughman, R. H.; Chen, Y. Three-Dimensionally Bonded Spongy Graphene Material with Super Compressive Elasticity and near-Zero Poisson's Ratio. *Nat. Commun.* **2015**, *6* (1), 6141. https://doi.org/10.1038/ncomms7141.
(10) Barg, S.; Perez, F. M.; Ni, N.; do Vale Pereira, P.; Maher, R. C.; Garcia-Tuñon, E.; Eslava, S.; Agnoli, S.; Mattevi, C.; Saiz, E. Mesoscale Assembly of Chemically Modified Graphene into Complex Cellular Networks. *Nat. Commun.* **2014**, *5* (1), 4328. https://doi.org/10.1038/ncomms5328.
(11) Liu, T.; Huang, M.; Li, X.; Wang, C.; Gui, C.-X.; Yu, Z.-Z. Highly Compressible Anisotropic Graphene Aerogels Fabricated by Directional Freezing for Efficient Absorption of Organic Liquids. *Carbon N. Y.* **2016**, *100*, 456–464. https://doi.org/10.1016/j.carbon.2016.01.038.
(12) Qiu, L.; Huang, B.; He, Z.; Wang, Y.; Tian, Z.; Liu, J. Z.; Wang, K.; Song, J.; Gengenbach, T. R.; Li, D. Extremely Low Density and Super-Compressible Graphene Cellular Materials. *Adv. Mater.* **2017**, *29* (36), 1701553. https://doi.org/10.1002/adma.201701553.
(13) Lin, Y.; Liu, F.; Casano, G.; Bhavsar, R.; Kinloch, I. A.; Derby, B. Pristine Graphene Aerogels by Room-Temperature Freeze Gelation. *Adv. Mater.* **2016**, *28* (36), 7993–8000. https://doi.org/10.1002/adma.201602393.
(14) Qiu, L.; Liu, J. Z.; Chang, S. L. Y.; Wu, Y.; Li, D. Biomimetic Superelastic Graphene-Based Cellular Monoliths. *Nat. Commun.* **2012**, *3* (1), 1241. https://doi.org/10.1038/ncomms2251.
(15) Zhu, C.; Han, T. Y.-J.; Duoss, E. B.; Golobic, A. M.; Kuntz, J. D.; Spadaccini, C. M.; Worsley, M. A. Highly Compressible 3D Periodic Graphene Aerogel Microlattices. *Nat. Commun.* **2015**, *6* (1), 6962. https://doi.org/10.1038/ncomms7962.
(16) Ni, N.; Barg, S.; Garcia-Tunon, E.; Macul Perez, F.; Miranda, M.; Lu, C.; Mattevi, C.; Saiz, E. Understanding Mechanical Response of Elastomeric Graphene Networks. *Sci. Rep.* **2015**, *5* (1), 13712. https://doi.org/10.1038/srep13712.
(17) Li, Y.; Chen, J.; Huang, L.; Li, C.; Hong, J.-D.; Shi, G. Highly Compressible Macroporous Graphene Monoliths via an Improved Hydrothermal Process. *Adv. Mater.* **2014**, *26* (28), 4789–4793. https://doi.org/10.1002/adma.201400657.
(18) Lv, L.; Zhang, P.; Cheng, H.; Zhao, Y.; Zhang, Z.; Shi, G.; Qu, L. Solution-Processed

Ultraelastic and Strong Air-Bubbled Graphene Foams. *Small* **2016**, *12* (24), 3229–3234. https://doi.org/10.1002/smll.201600509.
(19) Zhang, R.; Chen, Q.; Zhen, Z.; Jiang, X.; Zhong, M.; Zhu, H. Cellulose-Templated Graphene Monoliths with Anisotropic Mechanical, Thermal, and Electrical Properties. *ACS Appl. Mater. Interfaces* **2015**, *7* (34), 19145–19152. https://doi.org/10.1021/acsami.5b04808.
(20) Estevez, L.; Kelarakis, A.; Gong, Q.; Da'as, E. H.; Giannelis, E. P. Multifunctional Graphene/Platinum/Nafion Hybrids via Ice Templating. *J. Am. Chem. Soc.* **2011**, *133* (16), 6122–6125. https://doi.org/10.1021/ja200244s.
(21) Srinivas, G.; Burress, J. W.; Ford, J.; Yildirim, T. Porous Graphene Oxide Frameworks: Synthesis and Gas Sorption Properties. *J. Mater. Chem.* **2011**, *21* (30), 11323. https://doi.org/10.1039/c1jm11699a.
(22) Worsley, M. A.; Olson, T. Y.; Lee, J. R. I.; Willey, T. M.; Nielsen, M. H.; Roberts, S. K.; Pauzauskie, P. J.; Biener, J.; Satcher, J. H.; Baumann, T. F. High Surface Area, Sp 2 -Cross-Linked Three-Dimensional Graphene Monoliths. *J. Phys. Chem. Lett.* **2011**, *2* (8), 921–925. https://doi.org/10.1021/jz200223x.
(23) Zhao, X.; Hayner, C. M.; Kung, M. C.; Kung, H. H. Flexible Holey Graphene Paper Electrodes with Enhanced Rate Capability for Energy Storage Applications. *ACS Nano* **2011**, *5* (11), 8739–8749. https://doi.org/10.1021/nn202710s.
